# Midtrimester cervical elastography in pregnant women with a history of loop electrosurgical excision procedure (LEEP)

**DOI:** 10.1038/s41598-022-13170-9

**Published:** 2022-06-02

**Authors:** Hyun-Hwa Cha, Won Joon Seong, Hyun Mi Kim, Hyun-Joo Seol, Ji-Hee Sung, Hyun Soo Park, Han-Sung Hwang, Hayan Kwon, Yun Ji Jung, Ja-Young Kwon, Soo-young Oh

**Affiliations:** 1 Department of Obstetrics and Gynecology, School of Medicine, Kyungpook National University Chilgok Hospital, Kyungpook National University, Daegu, Korea; 2 Department of Obstetrics and Gynecology, Kyung Hee University School of Medicine, Seoul, Korea; 3 Department of Obstetrics and Gynecology, Samsung Medical Center, Sungkyunkwan University School of Medicine, 81 Irwon-ro, Gangnam-gu, Seoul, 06351 Korea; 4 Department of Obstetrics and Gynecology, Graduate School of Medicine, Dongguk University, Goyang, Korea; 5 Department of Obstetrics and Gynecology, Konkuk University Medical Center, Konkuk University School of Medicine, Seoul, Korea; 6 Department of Obstetrics and Gynecology, Institute of Women’s Life Medical Science, Yonsei University College of Medicine, Seoul, Korea

**Keywords:** Ultrasonography, Diseases, Medical research, Risk factors

## Abstract

We aimed to compare cervical elastographic parameters based on a previous loop electrosurgical excision procedure (LEEP) and to determine whether they can predict preterm delivery in pregnant women with a history of LEEP. This multicenter prospective case–control study included 71 singleton pregnant women at 14–24 weeks of gestation with a history of LEEP and 1:2 gestational age-matched controls. We performed cervical elastography using E-cervix and compared maternal characteristics, delivery outcomes, cervical length (CL), and elastographic parameters between the two groups. The median mid-trimester CL was significantly shorter in the LEEP group. Most elastographic parameters, including internal os (IOS), external os (EOS), elasticity contrast index (ECI), and hardness ratio (HR), were significantly different in the two groups. In the LEEP group, the sPTD group compared to the term delivery (TD) group showed a higher rate of previous sPTD (50% vs. 1.7%, *p* < *0.001*), higher IOS and ECI (IOS: 0.28 [0.12–0.37] vs. 0.19 [0.10–0.37], *p* = *0.029*; ECI: 3.89 [1.79–4.86] vs. 2.73 [1.48–5.43], *p* = 0.019), and lower HR (59.97 [43.88–92.43] vs. 79.06 [36.87–95.40], *p* = 0.028), but there was no significant difference in CL (2.92 [2.16–3.76] vs. 3.13 [1.50–3.16], *p* = 0.247). In conclusion, we demonstrated that a history of LEEP was associated with a change in cervical strain measured in mid-trimester as well as with CL shortening. We also showed that cervical elastography can be useful in predicting sPTD in pregnant women with previous LEEP.

## Introduction

The loop electrosurgical excision procedure (LEEP), a treatment modality for preinvasive cervical disease, is widely used globally, especially in women of reproductive age^1–3^. Although several studies have shown that LEEP is associated with an increased risk of spontaneous preterm delivery (sPTD) in subsequent pregnancies^4–6^, the direct pathogenesis of sPTD after LEEP remains unclear. Several reports have demonstrated that the mid-trimester cervical length (CL) in women with a history of LEEP is significantly shorter than that in women without such history^7–10^. However, a recent study reported no CL shortening or decreased cervical volume at 3–6 months after LEEP^11^. Therefore, predicting sPTD using CL alone in women with a history of LEEP seems to be limited.

Elastography has recently emerged as a promising ancillary tool to conventional ultrasound since it enables assessment of the mechanical properties of tissue and the stiffness as a strain value of the organs, including the breast and liver^12,13^. Effort is underway worldwide to introduce cervical elastography in the obstetric area to evaluate the cervix during pregnancy^14–20^. Studies have shown that cervical elastography could be an effective tool for assessment of women at risk of sPTD^15,21–27^. A recent study including non-pregnant women showed that the elasticity score measured by the 4-point elastography scoring system was significantly lower (i.e., indicating a softer cervix) after LEEP than before^28^. However, data assessing the change in cervical strain by elastography in pregnant women with a history of LEEP in relation to pregnancy outcomes are limited.

With this background, we aimed to determine whether there are differences in cervical elastography parameters and CL measured in mid-trimester pregnancies with a history of LEEP and to test whether these elastographic parameters could help predict sPTD in women with a history of LEEP.

## Results

### Maternal characteristics and delivery outcomes

During the study period, 71 women with singleton pregnancies and a history of LEEP and twice the number of women with no history of LEEP were recruited. Table 1 shows the maternal characteristics and delivery outcomes for participants in the LEEP versus control groups. Maternal baseline characteristics of age, pre-pregnancy BMI, nulliparity rate, abortion history, and sPTD did not differ between groups. However, progesterone treatment and cerclage operation were more frequent in the LEEP group. For delivery outcomes, the overall preterm delivery rate did not differ significantly between the two groups, but the rate of sPTD was significantly higher in the LEEP group.

**Table 1:** Maternal characteristics and delivery outcomes of the LEEP group versus the control group.

Maternal characteristics	LEEP group (*n* = 71)	Control group (*n* = 142)	*p* value
Age (years)*	33.9 ± 3.8	34.2 ± 3.8	0.582
Pre-pregnancy BMI^†^	22.5 (17.3–35.3)	22.4 (17.2–41.3)	0.966
Nulliparity (%)	37 (52.1)	78 (54.9)	0.697
History of abortion (%)	21 (29.6)	45 (31.7)	0.777
Spontaneous (%)	16 (22.5)	31 (21.8)	0.501
Artificial (%)	5 (7.0)	14 (19.9)	0.472
History of sPTD (%)	6 (8.5)	5 (3.5)	0.252
Progesterone after examination (%)	25 (35.2)	10 (7.0)	< 0.001
Cerclage after examination (%)	4 (5.6)	–	0.017
GA at elastography (weeks)^†^	18.5 (14.0–24.0)	17.6 (16.3–23.6)	0.831

Delivery outcomes	LEEP group^‡^ (*n* = 69)	Control group (*n* = 142)	*p* value
GA at delivery (weeks)*	37.8 ± 2.0	38.3 ± 2.2	0.095
Preterm delivery (%)	11 (15.9)	12 (8.5)	0.073
Spontaneous (%)	8 (11.6)	5 (3.5)	0.032
Indicated (%)	3 (4.3)	7 (4.9)	1.000
Birth weight (g)*	3102 ± 571	3193 ± 551	0.267
Cesarean section (%)	39 (56.5)	77 (54.2)	0.625

*Mean ± standard deviation; ^†^median (range); ^‡^excludes two cases of follow-up loss.

*BMI* body mass index, *GA* gestational age, *LEEP* loop electrosurgical excision procedure, *sPTD* spontaneous preterm delivery.

### Cervical length and elastographic parameters

Table 2 compares CL and cervical elastographic parameters between the two groups. The median CL was significantly shorter in the LEEP group versus the control group (3.03 [1.50–4.66] cm vs. 3.60 [2.60–5.29] cm, *p* < 0.001). The IOS and EOS were significantly higher in the LEEP group than in the control group (IOS: 0.20 [0.10–0.37] vs. 0.17 [0.05–0.39], *p* < 0.001; EOS: 0.25 [0.11–0.57] vs. 0.21 [0.09–0.49], *p* = 0.001). Moreover, the median ECI value, which reflects strain heterogeneity, was also higher in the LEEP group (2.81 [1.48–5.43] vs. 2.36 [1.04–4.70], *p* = 0.015). Last, HR, which reflects the upper 30th percentile hardness area ratio, was significantly lower in the LEEP group than in the control group (78.95 [36.87–95.40] vs. 85.48 [39.33–97.44], *p* = 0.001).

**Table 2:** Mid-trimester cervical length and cervical elastography parameters of the LEEP group versus the control group.

	LEEP group (*n* = 71)	Control group (*n* = 142)	*p* value
CL (cm)*	3.03 (1.50–4.66)	3.60 (2.60–5.29)	< 0.001
Short CL (%)	17 (23.9)	9 (6.3)	< 0.001
Funneling (%)	1 (1.4)	–	0.333
Elastography parameters*			
IOS	0.20 (0.10–0.37)	0.17 (0.05–0.39)	< 0.001
EOS	0.25 (0.11–0.57)	0.21 (0.09–0.49)	0.001
I/E ratio	0.78 (0.38–2.25)	0.76 (0.27–1.87)	0.580
ECI	2.81 (1.48–5.43)	2.36 (1.04–4.70)	0.015
HR (%)	78.95 (36.87–95.40)	85.48 (39.33–97.44)	0.001

*Median (range).

*CL* cervical length, *ECI* elasticity contrast index, *EOS* mean external os strain, *HR* hardness ratio, *IOS* internal os strain, *LEEP* loop electrosurgical excision procedure.

### Maternal characteristics and delivery outcomes in the LEEP group

In the LEEP group, after exclusion of two cases that were lost to follow-up and three cases of indicated preterm delivery, there were eight cases of sPTD. Table 3 compares maternal characteristics and delivery outcomes between the sPTD and TD cases in the LEEP group. Notably, the previous sPTD rate was significantly higher in the sPTD group (50% vs. 1.7%, *p* < 0.001). Progesterone treatment after CL measurement and elastography was more frequent in the sPTD group than in the TD group (75.0% vs. 27.6%, *p* = 0.014) because of the history of sPTD and a short CL. Overall, in the LEEP group, the cerclage rate was quite low (4 of 66 [6%]) and was similar between the sPTD and TD groups.

**Table 3:** Maternal characteristics and delivery outcomes of spontaneous preterm delivery versus term delivery in the LEEP group.

	sPTD group (*n* = 8)	TD group (*n* = 58)	*p* value
**Maternal characteristics**^†^			
Age (years)*	33.8 ± 3.8	34.2 ± 3.8	0.759
Pre-pregnancy BMI^‡^	21.0 (19.7–28.7)	22.7 (17.3–35.3)	0.268
Nulliparity (%)	3 (37.5)	32 (55.2)	0.459
History of abortion (%)	2 (25)	18 (31.0)	1.000
Spontaneous (%)	2 (25)	13 (31.0)	0.797
Artificial (%)	–	5 (8.6)	0.389
History of sPTD (%)	4 (50)	1 (1.7)	< 0.001
Progesterone after examination (%)	6 (75.0)	16 (27.6)	0.014
Cerclage after examination (%)	1 (12.5)	3 (5.2)	0.485
GA at elastography (weeks)^‡^	17.3 (14.0–21.0)	19.8 (14.0–24.0)	0.160
**Delivery outcomes**^†^			
GA at delivery (weeks)*	34.6 ± 2.4	38.3 ± 1.0	0.002
Birth weight (g)*	2408 ± 510	3225 ± 738	0.009
Cesarean section (%)	3 (37.5)	33 (56.9)	0.444

^†^Excludes three cases of iatrogenic preterm delivery; *mean ± standard deviation; ^‡^median (range).

*BMI* body mass index, *GA* gestational age, *LEEP* loop electrosurgical excision procedure, *sPTD* spontaneous preterm delivery, *TD* term delivery.

### Cervical length and elastographic parameters in the LEEP group

Table 4 compares CL and cervical elastographic parameters between the sPTD and TD cases in the LEEP group. There was no difference in CL between sPTD and TD in the LEEP group (2.92 [2.16–3.76] vs. 3.13 [1.50–3.16], *p* = 0.247). Of note, the sPTD group was associated with higher IOS and ECI (0.28 [0.12–0.37] vs. 0.19 [0.10–0.37], *p* = 0.029 and 3.89 [1.79–4.86] vs. 2.73 [1.48–5.43], *p* = 0.019, respectively) and a lower HR (59.97 [43.88–92.43] vs. 79.06 [36.87–95.40], *p* = 0.028).

**Table 4:** Mid-trimester cervical length and cervical elastography parameters of spontaneous preterm delivery versus term delivery in women with a history of LEEP.

	sPTD group (*n* = 8)	TD group (*n* = 58)	*p* value
CL (cm)*	2.92 (2.16–3.76)	3.13 (1.50–3.16)	0.247
Short CL (%)	4 (50.0)	12 (20.7)	0.090
Funneling (%)	1 (12.5)	–	0.121
**Elastography parameters**			
IOS	0.28 (0.12–0.37)	0.19 (0.10–0.37)	0.029
EOS	0.32 (0.15–0.46)	0.25 (0.11–0.27)	0.130
I/E ratio	0.70 (0.57–1.48)	0.79 (0.38–2.25)	0.937
ECI	3.89 (1.79–4.86)	2.73 (1.48–5.43)	0.019
HR (%)	59.97 (43.88–92.43)	79.06 (36.87–95.40)	0.028

*Median (range).

*CL* cervical length, *ECI* elasticity contrast index, *EOS* external os strain, *HR* hardness ratio, *IOS* internal os strain, *LEEP* loop electrosurgical excision procedure, *sPTD* spontaneous preterm delivery, *TD* term delivery.

### Receiver operating characteristics curve of predicting spontaneous preterm delivery in the LEEP group

**Figure 1:**
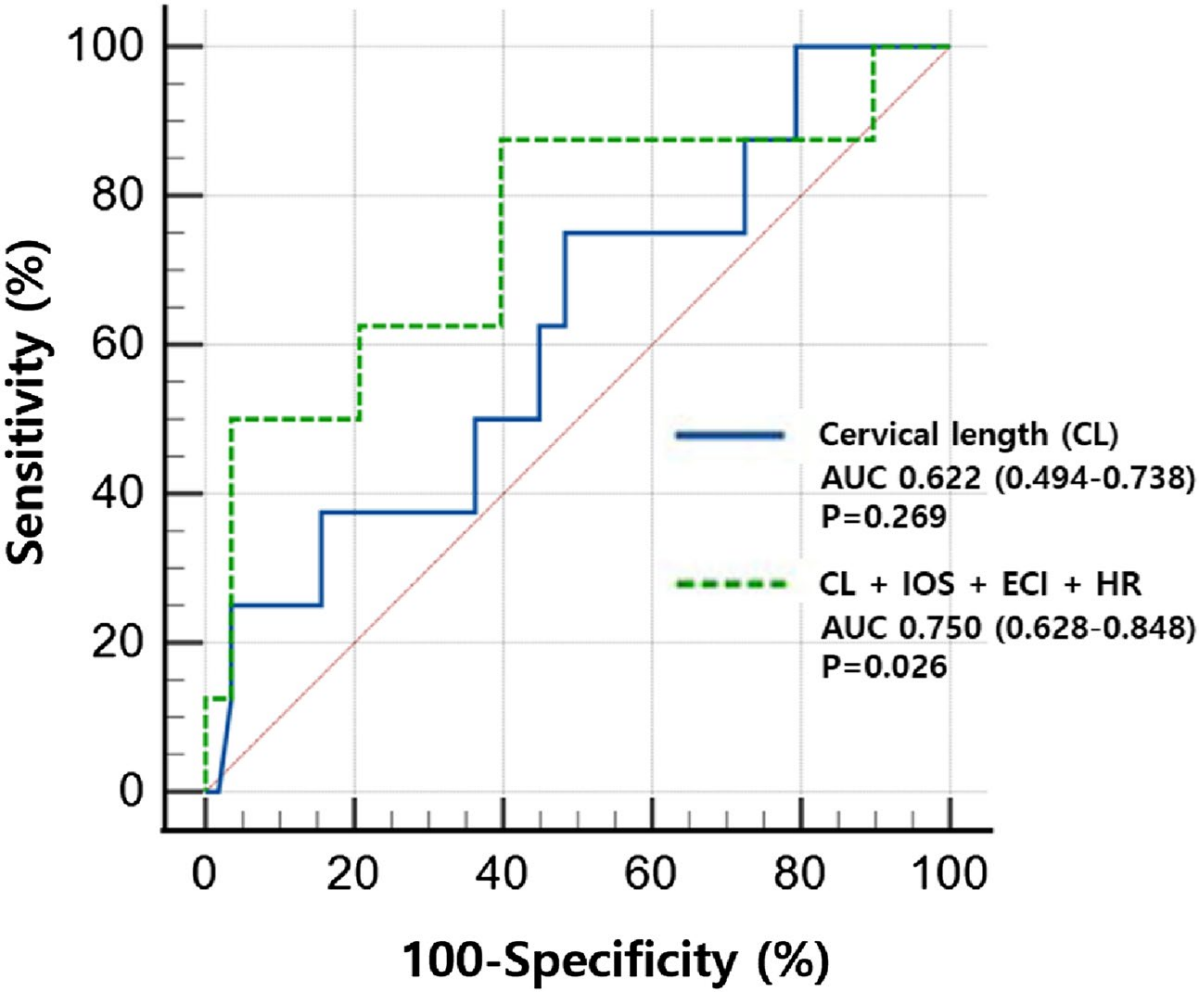
Receiver operating characteristic curve for predicting spontaneous preterm delivery in women with a history of LEEP using CL alone versus using CL and elastographic parameters of IOS, ECI, and HR. *CL* cervical length, *ECI* elasticity contrast index, *HR* hardness ratio, *IOS* mean internal os strain, *LEEP* loop electrosurgical excision procedure.

Table 5 shows the diagnostic performance of CL and elastographic parameters for predicting sPTD in the LEEP group by area under the ROC curve (AUC) analysis. The AUC of the CL was not statistically significant for predicting sPTD. However, IOS, ECI, and HR were able to predict sPTD with statistical significance. We found that the Youden index, a function of both sensitivity and specificity that is used to estimate the effectiveness of diagnostic criteria^29^, of the elastographic parameters (IOS, ECI, and HR) was higher than that of CL. We also noted that, although the sensitivities of CL and the elastographic parameters were similar, the specificities of the latter (IOS, ECI, and HR) were higher. Finally, we presented a predictive model for sPTD, consisting of CL, IOS, ECI, and HR. As shown in Fig. 1, the model was predictive of sPTD in the LEEP group (AUC = 0.750; 95% confidence interval [CI]: 0.628–0.848; *p* = 0.026), whereas CL alone was not (AUC = 0.622; 95% CI: 0.494–0.738; *p* = 0.269). However, this combined model failed to demonstrate a significantly higher AUC than CL alone in our study population (*p* = 0.395).

**Table 5:** Diagnostic performance of CL and elastographic parameters for predicting sPTD in the LEEP group by area under the ROC curve analysis.

Model	AUC (95% CI)	*p* value*	Youden index	Associated criterion	Sensitivity (95% CI)	Specificity (95% CI)
CL	0.622 (0.494–0.738)	0.269	0.267	≤ 3.03	75.0 (34.9–96.8)	51.7 (38.2–65.0)
IOS	0.739 (0.616–0.840)	0.042	0.453	> 0.26	62.5 (24.5–91.5)	82.8 (70.6–91.4)
EOS	0.666 (0.539–0.777)	0.135	0.323	> 0.24	87.5 (47.3–99.7)	44.8 (31.7–58.5)
ECI	0.756 (0.635–0.854)	0.012	0.560	> 3.49	75.0 (34.9–96.8)	81.0 (68.6–90.1)
HR	0.741 (0.619–0.841)	0.035	0.522	≤ 63.57	62.5 (24.5–91.5)	89.7 (78.8–96.1)

*AUC* area under the ROC curve, *CI* confidence interval, *CL* cervical length, *ECI* elasticity contrast index, *EOS* mean external os strain, *HR*, hardness ratio, *IOS* mean internal os strain, *LEEP* loop electrosurgical excision procedure, *ROC* receiver operating characteristic, *sPTD* spontaneous preterm delivery, *p value < 0.05 reflects statistical significance in predicting sPTD.

## Discussion

To the best of our knowledge, this is the first study to demonstrate changes in the cervical strain values measured in the mid-trimester in pregnant women with a history of LEEP. Specifically, multiple elastographic parameters, including IOS, EOS, ECI, and HR, as well as CL, showed significant differences in pregnant women with a history of LEEP versus controls. We also found a significant difference in multiple elastographic parameters between the sPTD and TD women in the LEEP group. Finally, the ROC analysis results suggested that the AUC values of IOS, ECI, and HR were able to predict sPTD with improved specificity compared to that of CL.

Although pregnancies in women with a previous history of LEEP are at increased risk of preterm birth, the exact pathogenesis of sPTD after LEEP is not clearly understood. Previous data suggest that the risk of sPTD is not merely due to shortening of the CL after this procedure^30^. It was also hypothesized that the increased sPTD risk might be attributable to cervical weakening or the underlying risk factors associated with cervical dysplasia, such as human papilloma virus infection, or alterations in vaginal flora and immune modulation^5^. Our current findings add evidence that the increased risk of sPTD after LEEP might be attributed to the change in cervical strain. These observations are in line with the recent opinion that the regenerative process after LEEP is related to cervical elasticity changes^28^.

Our result of CL shortening after LEEP corroborates the findings of previous studies^7–10^. These reports demonstrated that mid-trimester CL in women with a history of LEEP is shortened by 4–7 mm compared to women without a history of LEEP, which was similar to our result (5.7 mm). However, the association between CL shortening and sPTD in women with a history of LEEP remains controversial. Berghella suggested that detection of a short CL could be a predictive tool for sPTD based on 109 women who underwent prior cone biopsy^9^. In contrast, Parikh et al. showed that, although a shortened cervix was observed in 4.5% of patients in the LEEP group, no cases of sPTD occurred among these women^10^. In our study, 23.9% of patients in the LEEP group had a short CL, and 11.6% delivered spontaneously before 37 weeks of gestation. However, there was no difference in mid-trimester CL between sPTD and TD women in the LEEP group, and the AUC for prediction of sPTD was 0.622, indicating a low predictive value for CL.

Meanwhile, opening of the IOS is known to be associated with sPTD^31^, implying that IOS stiffness is more important than EOS characteristics in the maintenance of pregnancy. In fact, several studies have shown the role of IOS in predicting sPTD using various types of cervical elastography, including shear-wave elastography^22^, strain elastography^16,32^, and color mapping^17,21^. These studies included low-risk patients^16,17^, high-risk patients with a short CL^21^, or unselected cohorts^22,23^ and reported similar results for the association between IOS and sPTD. In our study, we targeted specific subjects with a history of LEEP to analyze the effectiveness and identified an increased IOS strain in sPTD versus TD.

In our study, EOS strain was significantly higher in the LEEP group than in the control group; however, there was no difference in this parameter between the sPTD and TD cases within the LEEP group. This implies that IOS strain is more important than EOS strain in predicting preterm delivery among women with a history of LEEP. It is also notable that the overall EOS strain in the control group as well as in the LEEP group seems higher than that of the IOS strain, reflecting a softer external os than internal os. This finding is compatible with the previous observation by Molina et al., who showed that the internal and inferior parts of the cervix were stiffer than the external and superior parts on cervical elastography^33^. Indeed, the difference in IOS and EOS strains could be explained by the difference in cellular and stromal components. For example, the area of the cervical EOS contains 10% smooth muscle cells, whereas the IOS contains 50–60%^34^.

Our study also demonstrated that the elastographic parameters of ECI and HR were significantly different between the sPTD and TD groups of the LEEP group. ECI and HR were also recently identified as important parameters for predicting sPTD^14,15,23^. Patberg et al. reported that an increasing ECI at 18–22 weeks was an independent risk factor for sPTD in singleton pregnancies^14^. Park et al. suggested that addition of ECI might improve the sPTD predictive ability in women with a CL of 1.5–2.5 cm^23^. A lower HR was associated with sPTD in women with threatened preterm labor^15^.

Our study has several limitations. Although this was a multicenter trial, we included only 71 pregnant women with a history of LEEP. Of them, sPTD occurred in only 8. However, the occurrence of sPTD was similar to the 9.4% of a previous study of 468 nulliparous singleton pregnancies with a history of LEEP^35^. It should also be mentioned that it is difficult to represent all pregnant women who underwent the LEEP procedure. The prevalence of a short CL (< 2.5 cm) was significantly higher in our LEEP population (23.9%) than in those of other studies (5.9%)^7^. Despite these limitations, our study has strength and originality as a multicenter prospective study that targeted a specific type of high-risk pregnancy in women with a history of LEEP.

In conclusion, we demonstrated that a history of LEEP was associated with a change in cervical strain measured by cervical elastography as well as CL shortening. Specifically, higher strain values of IOS, EOS, and ECI and lower values of HR reflecting the softness and heterogeneity of cervical strain were observed in pregnant women with a history of LEEP compared to the controls. Within the LEEP group, significant differences were noted in cervical strain including IOS, ECI, and HR but not in CL. Our data suggest that cervical elastography has the potential to improve the diagnostic predictive power for sPTD, especially in the direction of increasing specificity in pregnant women with a history of LEEP. We expect that the implementation of cervical elastography will be helpful in the management of pregnant women at risk for sPTD, such as those with a history of LEEP. Further studies including larger numbers of pregnant women with a history of LEEP are warranted.

## Methods

### Study design and participants

This prospective case–control study was performed by the Korean Research Group of Cervical Elastography, which included seven institutions (Kyung Hee University Hospital at Gangdong, Kangbuk Samsung Hospital, Kyungpook National University Chilgok Hospital, Dongguk University Ilsan Hospital, Konkuk University Medical Center, Yonsei University Severance Hospital, and Samsung Medical Center), between June 2018 and December 2020. According to the study protocol, which has been previously described^36^, we recruited women at 14–24 weeks of gestation with a history of LEEP as one of the study groups (LEEP group) for this multicenter study. For the control group without a history of LEEP, we selected twice the number of singleton pregnancies of matched gestational age (GA). This study was approved by the institutional review boards (IRB) of all participating institutions (IRB nos. Yonsei University Severance Hospital, 1-2018-0022; Dongguk University Ilsan Hospital, 2017-12-019-007; Kangbuk Samsung Hospital, 2018-06-006; Samsung Medical Center, 2018-03-073-015; Kyung Hee University Hospital at Gangdong, 2018-03-002; Kyungpook National University Chilgok Hospital, 2018-08-005-007H; and Konkuk University Medical Center, 1040070), and written informed consent was obtained from all participants prior to enrollment. We followed the ethical standards for human experimentation established in the Declaration of Helsinki.

### Measurement of cervical elastography

**Figure 2:**
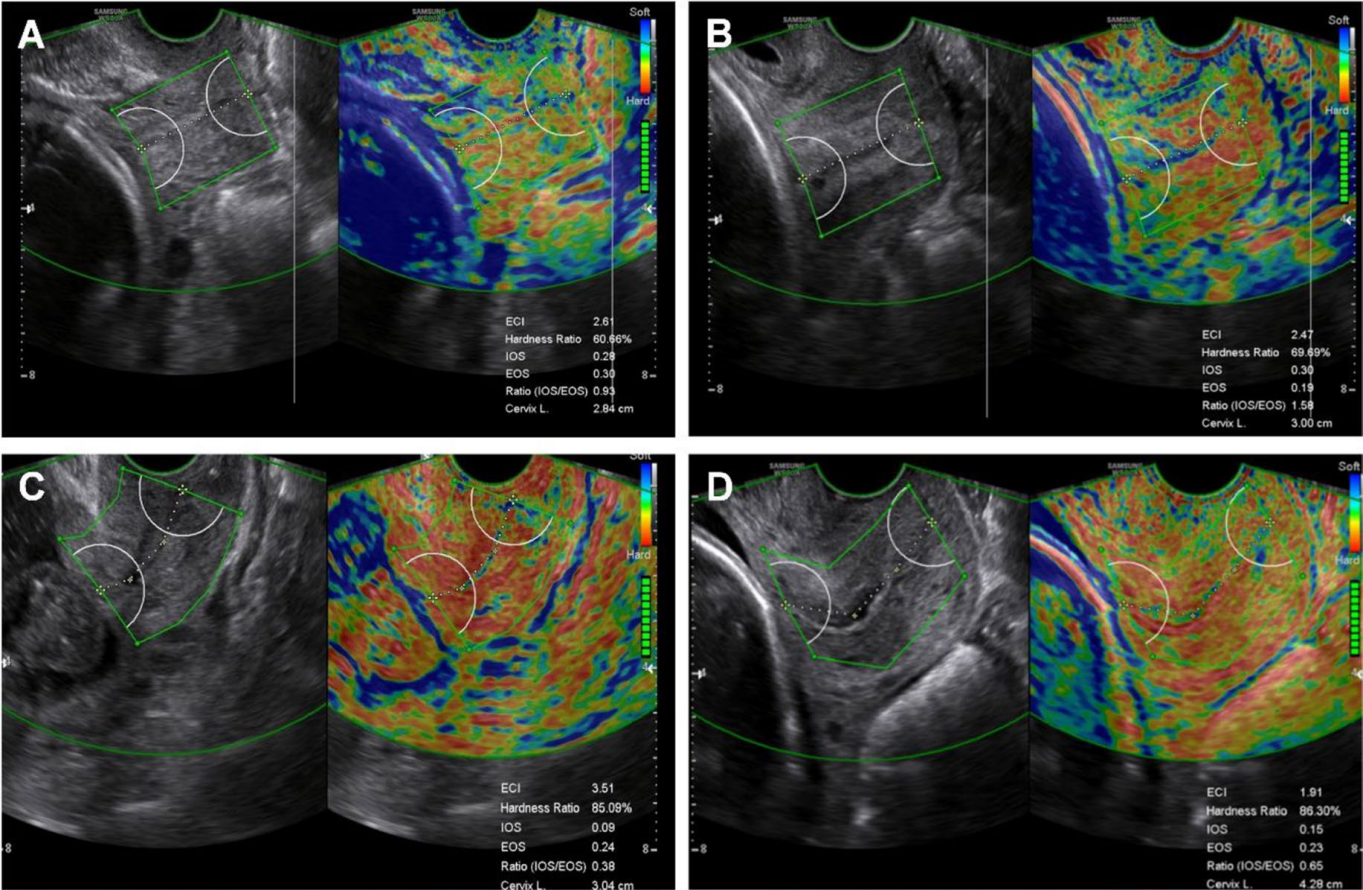
Images of cervical elastography using the E-cervix system. We identified the mid-sagittal plane of the cervix in which the endocervical canal is clearly delineated and the anterior width of the cervix is equal to the posterior width. Cervical elastogram in women in the LEEP group (**A**) and the control group (**B**) using 2-point ROI when the endocervical line is straight. Cervical elastogram in women in the LEEP group (**C**) and the control group (**D**) using 4-point ROI when the endocervical line is curved. *LEEP* loop electrosurgical excision procedure, *ROI* region of interest.

CL was measured with a vaginal ultrasound (WS80A Ultrasound System, Samsung Medison, Seoul, Korea), while cervical elastography was performed with an E-cervix system (Samsung Medison, Seoul, Korea) using a 6-MHz transvaginal probe in eligible patients, including cases and controls, at the time of enrollment. The measurement of cervical elastography was performed according to a standardized protocol (Fig. 2)^36^. During the elastography image acquisition process, the patient was allowed to breathe, and the probe was kept stationary without any pressure on the anterior cervix until the motion bars (reliability indicator) turned green, followed by an auto-freeze of the elastography image. For strain measurement, a region of interest caliper was placed on the internal os (IOS) and external os (EOS) using the 2- or 4-point option and then on each corner of the cervix. The E-cervix parameters were calculated automatically and immediately reported. The elastography parameters included in the analysis were the IOS and EOS of the cervix mean strain level, IOS/EOS (I/E) mean strain ratio, elasticity contrast index (ECI), and hardness ratio (HR)^23^. Elastography image acquisition was performed three times, and the mean values for each strain parameter were used for the analysis.

### Clinical variables and study populations

We collected data on baseline maternal characteristics of age, pre-pregnancy body mass index (BMI), parity, history of abortion, and sPTD. Information on the clinical course, including the rate of progesterone treatment and cerclage performance after cervical elastography, was noted. Delivery outcomes including GA at delivery, rate of sPTD (< 37 weeks), birth weight, and Cesarean section rate were also reviewed. We compared the baseline maternal characteristics, delivery outcomes, CL, short CL rate (defined as < 25 mm), and elastographic parameters between women with a history of LEEP and controls. Next, we compared these variables of sPTD and term delivery (TD) among women with a history of LEEP. Finally, we conducted a receiver operating characteristic (ROC) curve analysis to evaluate the performance of CL and cervical elastographic parameters in predicting sPTD in women with a history of LEEP.

### Statistical analysis

Variables were compared between groups using the Mann–Whitney U test, Pearson’s X^2^ test, and Fisher’s exact test as appropriate. ROC curve analysis was performed, and statistical analyses were performed using IBM SPSS ver. 26.0 (IBM Corp., Armonk, NY, USA) and MedCalc (MedCalc Software Ltd., Ostend, Belgium). Statistical significance was set at *p* < 0.05.

## Data Availability

The datasets generated or analyzed during the current study are not publicly available due to the privacy policy but are available from the corresponding author on reasonable request.
